# Variational Free Energy and Economics Optimizing With Biases and Bounded Rationality

**DOI:** 10.3389/fpsyg.2020.549187

**Published:** 2020-11-06

**Authors:** Morten Henriksen

**Affiliations:** ^1^Ministry of Defence, Karup, Denmark; ^2^AAU Business School, The Faculty of Social Sciences, Aalborg University, Aalborg, Denmark

**Keywords:** optimizing behavior, behavioral economics, rational choice, variational free energy principle, neuroeconomics

## Abstract

The purpose of this paper is to offer a new framework for understanding action, optimization, and choice when applied to economic theory more generally. By drawing upon the concept known as the variational free energy principle, the paper will explore how this principle can be used to temper rational choice theory by re-formulating how agents optimize. The approach will result in agent behavior that encompasses a wide range of so-called cognitive biases, as seen in the scientific literature of behavioral economics, but instead of using these biases as further indications of market inefficiencies or market failures, the paper will likewise attempt to show the limits to which these biases can inform or critique standard economic theory. The paper therefore offers up a “middle of the road” approach, in which the neoclassical agent is not quite as “rational” as rational choice theory assumes, but at the same time, not quite as irrational as behavioral economics would often have us believe.

## Introduction

In the theory of optimal control learning, the goal is to select an action that maximizes some value function such that the preferred state of the world would manifest given an action. This can take the form $u_{t}^{*}=\arg m\,a\,x\,V\,\left(s_{t+1}|u_{t}\right)=\pi \,\left(s_{t}\right)$, where*u* is an action, *s* is a world state, and π is an optimized action, an equation very much comparable to Bellman’s optimality principle believed to be informed by Morgenstern and Neumann’s book *Theory of Games and Economic Behavior* from 1944 (von Neumann and Morgenstern, 1944; Friston et al., 2010).

In Bellman’s optimality principle, problems and objectives are more or less the same, meaning that problems are framed in terms of reaching an objective or a goal. This could be how to minimize costs or maximize profits or utility (Bellman, 1957), and from this optimality principle, expected utility theory follows quite naturally. We cannot, however, continue in this manner without reference to a predefined end state, in which the information contained or available at this state is presupposed. The goal is then to formalize the exact behavior that would facilitate a given optimal future condition. This behavior is given by the axioms of expected utility, whereby a strict adherence to these axioms allows the economist to use positive affine transformations on expected/subjective utility, giving utility itself an ordinal, if not cardinal value (Karni, 2014). As such, economics provides an axiomatic explanation to human action and presumes a set of behavioral laws on the basis of mathematical necessity/design.

This is very important, because it allows the economist, mathematician, or physicist to operationalize the immaterial or intensive, such as the subjective feeling of better and worse, into the material or extensive, such as the objective description of utility and disutility. The reason for this is that utilities are identical to positive affine transformations if, and only if, certain underlying axioms are not violated. We can see this by writing *au+b*, where *a* > 0, *u* is utility, and *b* is any real number. If *u*_1_ > *u*_2_, then it would also be the case that *au*_1_ + *b* > *au*_2_ + *b* as long as *a* is positive. Being able to represent utility numerically then places utility within a probability space, or σ-algebra, containing the collection of events that can be assigned probabilities as well as associated conditional probabilities.

### The Axioms of Expected and Subjective Utility

When utility is operationalized, then utility can be expressed in accordance with measure-theory, given that any real number exists within the space *ℝ^n^*. Concordantly, a real value function for *u* must be present, depending on the outcomes {*x*_1_,*x*_2_,…,*x_n_*}, and their relative probabilities (*x*_1_,*p*_1_;*x*_2_,*p*_2_;…;*x_n_*,*p_n_*). A number within *ℝ^n^* can then be thought of as a point within a space defined by set theory and expressed as a particular state or outcome belonging to a set of states or outcomes that is a superset of various sets of acts.

In economics, the underlying axioms (von Neumann and Morgenstern, 1947) are typically presented in the following way:

#### Completeness

A preference ordering is complete if and only if, for any two outcomes X and Y, an individual prefers X to Y, prefers Y to X, or is indifferent between the two. Formally this can be represented as (*x*_1_,*x*_2_) > (*y*_1_,*y*_2_)*and*(*x*_1_,*x*_2_) < (*y*_1_,*y*_2_)*and*(*x*_1_,*x*_2_)∼(*y*_1_,*y*_2_), with respect to a class of outcomes or a basket of goods.

#### Transitivity

For any three outcomes, X, Y, and Z, if X is preferred to Y, and Y is preferred to Z, then X must be preferred to Z as well. *if*(*x*_1_,*x*_2_) > (*y*_1_,*y*_2_)*and*(*y*_1_,*y*_2_) > (*z*_1_,*z*_2_)*then*(*x*_1_,*x*_2_) > (*z*_1_,*z*_2_).

#### Reflexivity

(*x*_1_,*x*_2_)≥(*x*_1_,*x*_2_), meaning that any outcome is at least as good as an identical outcome, or any good is at least as good itself.

#### Revealed Preferences

If it is revealed that outcome X is preferred to outcome Y, then it cannot be revealed that outcome Y is preferred to outcome X. In economics, we typically say that an individual will always choose X over Y if both choices are present on the budget line m so that*p*_1_*x*_1_ + *p*_2_*x*_2_ = *mandp*_1_*y*_1_ + *p*_2_*y*_2_ = *m*, unless relative prices change. Formally, this can be expressed as px(p,′m)′≤m∧x(p,′m)′≠x(p,m)⇒px′(p,m)>m′.

Given these axioms, consumer behavior can be expressed through indifference curves that illustrate the concept of consumer equilibrium. But more importantly, the axioms provide the conditions for a monotonic scale from which utility can be measured and expressed numerically (von Neumann and Morgenstern, 1947).

In addition to the four axioms listed, two more must be discussed as they, together with *completeness* and *transitivity*, are essential for the expected utility hypothesis. These two axioms are *independence* and *continuity*. Both *completeness* and *transitivity* deal with preference under risk, whereas the next two axioms deal with preferences under uncertainty (Savage, 1972).

#### Independence

Let *p* ∈ [0,1] and X, Y, and Z be independent outcomes or independent probability distributions over outcomes, so that a weak preference over X to Y occurs if and only if *pX* + (1−*p*)*Z* is weakly preferred to *pY* + (1−*p*)*Z*. This means that preferences over outcomes cannot be influenced by factors that are not relevant to the initial preference order. Suppose one is faced with the choice of two lotteries, (*p*)[*q*(*V*) + (1−*q*)(*U*)] + (1−*p*)(*Z*) and (*p*)(*Y*) + (1−*p*)(*Z*). Independence says that, if the first lottery is preferred over the second, then what determines this preference cannot have anything to do with the commonalities (*p*) and (1−*p*)(*Z*). The choice is thus solely a choice between *q*(*V*) + (1−*q*)(*U*) and Y. Embedding a direct lottery in a compound lottery, without changing the initial conditions, should therefore have no relevance in the valuation of the respective lotteries.

#### Continuity

Using the example from the independence axiom, continuity can be expressed as an indifference qualifier. If one prefers X to Y to Z, then there must exist a particular *p* ∈ [0,1] such that one is indifferent between the lottery *pX* + (1−*p*)*Z* and the certainty of Y. Continuity thus states that a unique probability distribution can always be found, such that one is indifferent between the probability of receiving the most preferable outcome, plus the probability of receiving the least preferable outcome, and the certainty of receiving the middle outcome (Levin, 2006).

The important takeaway through all this is that most optimization problems involving choice must implicitly accept a set of behavioral axioms that allows for positive affine transformations. Without the possibility for this transformation, a real value function for utility cannot exist, and the mapping of action between two states will consequently prove exceedingly difficult, if not impossible. In other words, we cannot measure utility given its non-material quality and therefore cannot in any normative sense consider a set of choices as optimal in comparison to any other set of choices. We can, however, measure a number attached to utility, but only if there exists an equivalency between optimal behavior, utility maximizing behavior, and mathematical optimization, naturally fostering a discussion about the degree to which optimal behavior is representative of actual human behavior. While there have been many attempts over the years to augment expected utility theory by removing or altering various axioms (Rényi, 1955; Aumann, 1962; Dubins, 1975; Giles, 1976; Giron and Rios, 1980; Fishburn, 1982; Blume et al., 1991; Galaabaatar and Karni, 2013; Zaffalon and Miranda, 2017), all of these alternative approaches to expected/subjective utility theory rely on a general axiomatic foundation for utility nonetheless, either directly or by approximation (i.e., lexicographic preferences/ordering).

This paper, far from criticizing the work of von Neumann and Morgenstern (1947), Savage (1972) and others, will, however, try to take a different approach to the concept of optimal behavior. By completely internalizing utility, the need to externalize utility through positive affine transformations is bypassed, likewise bypassing the need for an axiomatic foundation for optimal behavior. Here information alone will provide the connection between a partition of internal and external states though entropy or surprise and move the objective toward optimizing beliefs about world states, rather than the explicit utilities of world states. As a consequence, the ordering of preferences becomes “secondary” to the ordering of actions, by recognizing that the imperative is to reduce expected surprisal through adaptation. This will result in preferences that are highly context dependent and, while it will be correct, that at any given point in time a specific preference order exists (i.e., utility function); this preference order must be influenced by time expressed as surprisal/entropy in t + 1. The dynamic here presented also extends to continuity and the concept of indifference, as will be shown later.

### Why Variational Free Energy

The variational free energy principle is herein used, in order to provide a first principle account of how systems must behave given a very high degree of complexity. It does this by suggesting a variational approach to optimal behavior that absorbs both Bayesian decision theory (Berger, 2011) and optimal Bayesian design (Lindley, 1956). Here, the objective is to minimize variational free energy (Parr and Friston, 2019) and by this process provide a formal description of *bounded rationality* through the use of evidence bounds. By minimizing variational free energy, what is meant by this is simply the application of variational mathematics to Bayesian optimality, where free energy refers to the evidence bound between a recognition density and a generative density, also referred to as a Kullback–Leibler divergence. Minimizing this bound is therefore the same as minimizing system entropy, which again can be seen as a scheme for the maximization of model evidence. Variational free energy therefore casts an upper bound on entropy/surprise or a lower bound on model evidence/negative surprise, the minimization of which results in either the update of beliefs (change of internal states) or action (change of external states).

When Bayes optimality is subjected to a variational approach, bounded rationality simply becomes approximate Bayesian inference (Winn and Bishop, 2005), the foundational mechanism by which the process of active inference is achieved. Approximate Bayesian inference is a method that estimates the posterior distribution/density, due to the computational complexity associated with evaluating likelihood functions in complex problems. This method can therefore be used to formalize the concept of bounded rationality, as laid forth by Simon (1957), when bounded rationality is seen as a cognitive process limitation (Lebiere and Anderson, 2011). Given the emphasis on optimization under constraints, it may be argued that we are not strictly describing bounded rationality here, but *boundedly rationality* (Arrow, 2004). This argument will, however, encounter problems when recognizing that the imperative is to optimize beliefs rather than maximize expected utility explicitly. By recognizing the intractability of exact Bayesian inference given the level of complexity we are focused on, the variational approach, through free energy, defines an upper bound on surprise or a lower bound on model evidence/negative surprise. Action will hereby proceed in reference to a functional of probability distributions over preferred states, whereby current beliefs dictate the conditions for optimal behavior, due consideration for prior preferences taken (Friston et al., 2017; Parr and Friston, 2017). From the perspective of Bayesian decision theory (Berger, 2011), this allows us to utilize the complete class theorem (Brown, 1981) when referring to both subjective utility and prior preferences, equating one with the other.

The complete class theorem says that for any pair of loss functions and decisions, there are some priors that render the decision space optimal. This necessarily introduces a duality between loss functions and priors that is resolved by making them the same thing. In other words, for any observed choice or decision, there are some priors that render this decision Bayes optimal and, as such, described by the free energy principle. By coupling this with sensory information update, what could be termed a constrained optimization problem turns into an adaptive optimization problem. So even though prior preferences are called on to play the role of subjective utility, we are not in possession of an objective function that describes the “goal” of the optimization problem ex ante. In other words, we cannot say that the objective of an agent is to minimize variational free energy but only that the minimization of free energy provides the framework through which any objective must be reached. With the free energy formulation, we can therefore not prescribe an agent any specific objectives or values but only state that there must be an  objective function and that this function is controlled exclusively by beliefs. While this will render many statements derived from  the complete class theorem as unfalsifiable (Bowers and Davis, 2012), the theorem will primarily be leveraged to challenge the precision of various scientific statements invoking the predicates rational and irrational in regard to human behavior.

In the free energy principle, the goal is not to maximize expected utility or the value of a world state but to optimize beliefs about world states formally represented by $u_{t}^{*}=\arg m\,i\,n\,F\,\left(Q\,\left(s_{t+1}\right)|u_{t}\right)$ through subsequent actions $\pi^{*}=\arg\min\,\sum_{\tau }F\,\left(Q\,\left(s_{\tau }\right)|\pi \right)$ where *u*_τ_ = π(τ). Here, the first task will be to resolve uncertainty about the consequences of subsequent actions, which means that action cannot be a function of the states in the world but must be a function of beliefs about states in the world. This generates an intensity measure, or more precisely an energy functional, here the free energy function *F* of a function *Q* which describes an approximate posterior distribution indicating beliefs given a policy π. We are not just trying to optimize the next best action but the best sequences of actions in line with a path integral, or a time average ∑_τ_, of an energy functional of beliefs about future world states *s*_τ_. This is basically the same as invoking Hamilton’s principle of least action (i.e., accumulated cost) when framing policies in terms of good or bad behavior as conceptualized by the economist, read, rational, or irrational (Friston et al., 2012b). It is, however, important to note that this formulation places an emphasis on the order in which actions are undertaken.

The above equation says that the best way to move from one point another, with beliefs about world states given a particular policy, is to minimize variational free energy at each step. Hamilton’s principle of least action says that the best path between two points in space–time given a set of configurations (Lagrangian) is the path where action is stationary (least). Therefore, if we are in the business of minimizing free energy, “optimal behavior” can be construed as following the principle of least action, which, again, is a principle for minimizing the cost associated with performing an action. Placed in a policy framework, optimal behavior therefore implies ordered actions and not ordered preferences *per se*, shifting the impetus toward the context in which a preference becomes manifest by highlighting the cost associated with deviations from stationary action (i.e., adaptation).

Applying Hamilton’s principle of least action, in the context of an information theoretic treatment of self-organization, can be read as a tendency to resist an increase in disorder or entropy by minimizing surprise (Friston, 2013). This can be cast in terms of minimizing variational free energy that affords an upper bound on surprise. Crucially, because surprise is also negative log evidence, this looks exactly as if the system is trying to maximize the evidence for its model of the world. In other words, self-organization can be construed as self-evidencing (Hohwy, 2016) by the minimization of expected self-information. All of these perspectives are complementary ways of thinking about exactly the same underlying phenomenon; namely, organization to an attracting set of states that define the kind of states any system prefers to be in.

We will see later how axiomatic failures are generally due to context-sensitive choice behavior. This reflects a fundamental distinction between economics and the free energy principle. Because the free energy is a functional of probability distributions of beliefs, this means that optimal choices (under some particular prior preferences) depend upon current beliefs (Friston et al., 2017; Parr and Friston, 2017). This is important because it means that for any value of goods, the actual decisions will depend upon beliefs at the time of deciding. This means *there is no one-to-one mapping between choice behavior and prior preferences or subjective utility*. A key drive of this epistemic behavior is the value of information (Howard, 1966), reflecting the fact that the imperative is to reduce expected surprisal or divergence from prior preferences, not to maximize expected utility (Friston et al., 2012a). This difference is made formally apparent when one examines the various components of expected free energy that include expected utility, when subjective utility is associated with prior preferences.

### Variational Free Energy

The principle states that all organic systems are characterized by a common feature, the ability to combat the dispersal forces of entropy on the cellular level, and counterbalance entropy by exploiting energy from the surrounding environment. How organic systems do this is by minimizing the property known as variational free energy. An interesting aspect of this principle is its logical extension from a biological/thermodynamical principle, to a technical description of neurological processes. It is, however, necessary to point out that variational free energy is not to be confused with thermodynamic free energy. Under the free energy principle, variational free energy is the upper bound on entropic surprise, the surprise element being unforeseeable or atypical states and events confronting the agent. This is also known as the negative logarithm of model evidence in information theory, where the average surprise over time is entropy. More commonly, entropy is often interpreted as the law that all things move to a less and less ordered state, and while this interpretation is applicable herein, this paper will exclusively refer to Claude Shannon’s average surprise over time formulation whenever entropy is mentioned. More intuitively, we can view entropy as the average amount of information expected to be gained when sampling any random variable. In order to minimize variational free energy, the brain must generate a probabilistic model of the environment and all the events typically encountered within it (Friston et al., 2006; Buckley et al., 2017).

This model comprises a recognition density that corresponds to the posterior probability of the hidden causes of sensory input and a generative density that comprises a likelihood and prior. The likelihood is simply the probability that the sensory data were generated by the hidden states or causes, while priors correspond to prior beliefs about those causes before seeing the data. The Kullback–Leibler (KL) divergence between the recognition density (i.e., posterior) and the true posterior under the generative density is referred to as an evidence bound or variational free energy. By minimizing variational free energy, the KL divergence approaches zero and the free energy becomes the negative logarithm of model evidence. Instead of having a model of the environment generated from the ground up by sensory input alone, the free energy principle suggests that the sensory inputs from the environment are inferred initially from prior beliefs and subsequently matched with sensory input by active sampling, often referred to as active inference. This active inference generates prediction errors by matching predicted with actual signals, and through iterative sampling, i.e., iterative acts, the prediction errors diminish, minimizing the KL divergence along with variational free energy (Friston et al., 2006, 2009, 2010; Buckley et al., 2017). Behavior can thereby be described by the following equation that prescribes the probability of any policy in terms of expected free energy.

(1) ln⁡P⁢(π)=-G

where *ln*⁡*P*(π) is the probability distribution of a policy π, and G is the expected free energy. What we are saying here is simply that the selection of a policy has a cost and that this policy cost is equal to expected free energy. As such, this equation places a cost on cognition (Kool et al., 2010; Redish, 2013).

All policies will therefore be initiated with reference to the free energy the policy is expected to minimize:

(2) $$G=\sum_{\tau }E_{Q\,\left(O_{\tau },S_{\tau }|\pi \right)}\,\left[\ln P\,\left(O_{\tau },S_{\tau }|\pi \right)-\ln Q\,\left(S_{\tau }|\pi \right)\right]$$

where *ln*⁡*P*(*O*_τ_,*S*_τ_|π) is an energy term that describes that hidden states in the world *S*_τ_ cause observable outcomes *O*_τ_ given a particular policy π, and *P* is the probability distribution of outcomes that are generated by hidden states. The *ln*⁡*Q*(*S*_τ_|π) term describes the belief in the consequences of a policy, where *Q* is an approximate posterior distribution of this belief, here playing the role as the recognition density mentioned earlier. (Technically, a distribution is over discrete event spaces, while a density is over continuous random variables.) This can be rearranged in terms of risk sensitivity and subjective utility (Recall that we used the complete class theorem earlier in order to associate prior preferences with subjective utility.).

(3) $$G=E_{Q\,\left(O_{\tau },S_{\tau }|\pi \right)}\,\left[\ln Q\,\left(S_{t}|O_{\tau },\pi \right)+\ln P\,\left(O_{\tau }|m\right)-\ln Q\,\left(S_{\tau }|\pi \right)\right]$$

If we remove any uncertainty or ambiguity with regard to observations *ln*⁡*Q*(*S*_*t*_|*O*_τ_,π), then what is left will be the KL divergence, here indicating risk sensitivity or KL control *E*_*Q*(*O*_τ_,*S*_τ_|π)_[*ln*⁡*P*(*O*_τ_|*m*)−*ln*⁡*Q*(*S*_τ_|π)]. Without ambiguity, this term says that states are no longer hidden, which means that states and observations are the same. Here the KL divergence scores the difference between what we believe will happen given a particular policy *ln*⁡*Q*(*S*_τ_|π) and what we want to have happen *ln*⁡*P*(*O*_τ_|*m*) given a generative model of the world *m*, which can also be described as our prior preferences about long-term outcomes. This difference is therefore the same as a score for the objective risk we are willing to accept, assuming a subjectively well-defined log-likelihood. In other words, what is the perceived probability of ending up in a specific world state, and how badly do we want to do so.

This also means that if we remove relative risk *ln*⁡*Q*(*S*_τ_|π), what we are left with is a term describing subjective utility under risk/negative Bayesian risk *E*_*Q*(*O*_τ_|π)_[*ln*⁡*P*(*O*_τ_|*m*)], where we expect our actions to maximize the probability of ending up in a preferred world state based exclusively on our prior beliefs (notice that the equation contains no hidden states).

The expected free energy can also be rearranged to be expressed in terms of subjective utility under uncertainty (i.e., expected log evidence) and information gain. Here subjective utility is associated with prior preferences about long-term outcomes under the consideration of hidden states, where there is an attraction to states of high expected probability or minimum expected surprise. Because surprise is also negative log evidence, expected/subjective utility can be seen as the same as expected log evidence through the minimization of expected surprise/minimum expected deviation from prior preferences. The second term of Eq. 4 scores the information gain in terms of the divergence between beliefs about future states with and without observing outcomes. This is an important quantity in optimal Bayesian design (Lindley, 1956) and many other fields, where it is variously called salience, intrinsic value, intrinsic motivation, and value of information (Oudeyer and Kaplan, 2007; Itti and Baldi, 2009; Schmidhuber, 2010; Barto et al., 2013).

(4) $$G=E_{Q\,\left(O_{\tau },S_{\tau }|\pi \right)}\,\left[\ln P\,\left(O_{\tau }|m\right)\right]+E_{Q\,\left(O_{\tau }|\pi \right)}\,\left[D_{K\,L}\,\left[Q\,\left(S_{\tau }|O_{\tau },\pi \right)\,Q\,\left(S_{\tau }|\pi \right)\right]\right]$$

The last term *E*_*Q*(*O*_τ_|π)_[*D*_*KL*_[*Q*(*S*_τ_|*O*_τ_,π)*Q*(*S*_τ_|π)]] describes information gain that resolves uncertainty by scoring the degree to which uncertainty is reduced by pursuing a given policy (Friston et al., 2017, 2016; Parr and Friston, 2019). Here, the notion of adaptation is formally introduced, where the imposition of unexpected information generates the necessity for context-dependent belief updating.

If we then suggest that G can be viewed as an action scheme that scores the goodness of any action sequence or policy (π), we can then start to model an agent based on variational first principles.

## The Free Energy Agent

Much of economics rests upon temporal discounting and the change in the subjective value of various goods depending upon when they will be secured. As with the broader literature (Frederick et al., 2002; Kurth-Nelson and Redish, 2010), under the free energy (active inference) formulation, this time dependency is a natural consequence of dealing with uncertainty. In other words, if there is uncertainty about volatility or how states of the world unfold, then the expected utility of a particular outcome will decrease as it recedes into the future (Friston et al., 2014). This is a natural consequence of a loss of confidence (i.e., expected utility) about a preferred outcome that is a natural consequence of accumulating uncertainty (Schwartenbeck et al., 2015). Interestingly, time itself can be associated with the rate of belief updating that depends upon the precision of beliefs about the way things change (Mathys, 2012). This means there is an intimate relationship between time sensitive changes in valuation, and the precision of beliefs about state transitions. Here a likelihood mapping is in the generative model used to evaluate (expected) free energy. In what follows, the term “time perception” will be used to refer to the information gain, that is, the total amount of belief updating from the beginning of the action sequence to the end.

### Time Perception

Because belief updating is dependent on surprise/entropy, and the fact that entropy is not uniformly distributed, it is suggested that time perception is connected to the amount of belief updating/work that must be performed within an externally allotted time frame. This can be interpreted as an agent’s sense of urgency. So even though uncertainty about state transitions increases over time (Frederick et al., 2002; Kurth-Nelson and Redish, 2010), time perception will be influenced by the rate of belief updating that is required under various conditions. If conditions change unexpectedly, this will influence time perception/urgency (Roseboom et al., 2019), here a derivative of the cost of computation given entropy.

We therefore have a mechanism by which time perception (information gain) is matched with, and mediated by, expected free energy, communicating the degree to which an expected action sequence is frustrated given the need for adaptation. This is important, because it means that prior preferences, and the precision with which these prior preference are held (Pezzulo et al., 2018), directly determine the amount of entropy in the entire action sequence from beginning to end through well-defined expectations or beliefs (Schwartenbeck et al., 2013). These prior preferences reflect a time sequence between a felt want and expected utility at a future point in time, the divergence of which is composed of a series of expected sequences of actions (i.e., policies). When precision of prior preferences is written down as a function of time, expected free energy therefore reflects a time discount function that changes over time in response to various levels of uncertainty about state transitions (Kurth-Nelson et al., 2012).

### Temporal Discounting

The dynamics of the variational free energy principle means that active inference on future states (temporal discounting) will only partly conform to “external time” as both process limitations and adaptation becomes influencing factors. The aspect of the free energy formulation that may constitute a problem, however, is the inability of the principle to properly disentangle choice behavior from temporal choice behavior, as all choices will have an associated temporal dimension. Coupled with the complete class theorem, this means that we can make any perceivable discounting function conform to the principle, only with varying degrees of probability. Exponential (Samuelson, 1937), hyperbolic (Ainslie, 1991), and quasi hyperbolic (Laibson, 1997) discount functions would all be permissible strategies for any free energy agent, as well as the “speed” of the discounting rate. It will therefore be easy to fall into the trap of ex post reasoning, claiming that any new study on discounting behavior can be explained with reference to variational free energy. However, the key phrase is *varying degrees of probability*. The free energy formulation is an account from first principles on how systems must behave. By analyzing its different components, we therefore see the different conditions that must be present given a specific behavior. If agents’ discounting behaviors were conforming to an exponential function, then there would have to be very strict controls on the amount of information an agent could sample, or the time frame that the agent would have to “simulate,” eliminating the influence from the information gain term. In a real world situation, this would not be impossible, but highly improbable. If agents’ discounting behaviors were conforming to a hyperbolic function, then there would have to be a sufficiently large temporal dimension that would increase uncertainty (accumulated cost), to the point where further temporal increases become too costly to simulate, which is a lot more probable. In regard to the smaller sooner/larger later effect for instance, we should therefore be able to design a simple experiment that would make this effect disappear.

The hyperbolic discounter would prefer $5 now over $7 tomorrow, but not $5 in a year over $7 in a year and a day. In the free energy formulation, this preference reversal is due to the uncertainty/cost in dealing with small consequences over large time scales. What the agent is actually answering is therefore more like answering if he would prefer $5 now or $7 tomorrow, and $5 in a year or $7 in a year, simply evaluating the present value of the future $5 compared to the $7. To remove this effect, we would have to ask if the agent preferred $5 now or $7 tomorrow and then ask the agent to first imagine jumping 1 year forward in time and then proceed to repeat the question. This should keep preferences stable with a higher probability (Peters and Büchel, 2010; Benoit et al., 2011). As such, we are asking the same question, but restricting the amount of information the agent has to contend with by removing the cost/strain afforded by the temporal simulation. A way to quickly visualize this dynamic would be to envision a confidence interval growing larger and larger over time, as certainty about state transitions diminish proportionally. As this interval grows larger, the cost associated with deviations from least action will be diluted, as well as its influence on expected free energy evaluated in the present. The probability that a future choice simply reflects a discounted present choice will therefore be small. Discount rates will, however, change during the discounting period in response to information gain with a far higher probability, reflecting belief updating and a preceding search function (order) indicating the appropriateness of a given policy (uncertainty resolution) (Kurth-Nelson et al., 2012). This approach will hereby differ from models that explain temporal discounting in terms of temporal projection or future episodic thinking (Johnson and Redish, 2007; Kurth-Nelson et al., 2012; van der Meer et al., 2012) and will be more in line with suggestions from construal level theory (Trope and Liberman, 2010), where the goal is to figure out how to traverse a distance under various levels of abstraction, or as the free energy approach would put it, various degrees of uncertainty about state transitions (Schultz, 2016; Coddington and Dudman, 2017; Covey and Cheer, 2019). That being said, various efforts designed to reduce uncertainty over larger and larger time frames, such as temporal projection or episodic future thinking, should result in more stable discount functions due to a decrease in expected complexity associated with the simulation of state transitions (Peters and Büchel, 2010; Benoit et al., 2011; Kurth-Nelson et al., 2012).

Hopefully the connection between time perception and information gain has now become clearer. The crucial part is to disconnect “external time” from “internal time,” where the latter refers to the amount of work, or the strain of the system, responding to surprise/entropy. Changing external time frames will therefore not result in more or less “heuristic” behavior but will result in more or less urgent behavior. Here this urgency feeds back into expected free energy, possibly influencing preference ordering and discount functions. This “strain” is exactly the information gain, which follows from the fact that the information gain is mathematically the expected degree of belief updating as measured by the KL divergence in Eq. 4. The higher the cognitive strain, the lower the present value of more and more remote rewards, all else equal, and conversely, the faster the discount rate on the future (Ebert and Prelec, 2007; Peters and Büchel, 2010).

## Further Implications

Any action is initiated by a call for alleviation or a rectification of a psychological or biological state. The action schema (G) then posits a set of causally related action sequences or policies expected to work. The most often used successful strategies in the past convey the highest probability for success, resulting in a strongly weighted expectation following the principle of least resistance or Hamilton’s principle of least action, which under certain restrictive assumptions (e.g., perfect information) can also be expressed as KL control or risk sensitivity (van den Broek et al., 2010). The first step in any action sequence will be to search out the information that is predicted to indicate an appropriate policy (order). Based on the available information in the environment, each sequential step toward the desired end state is then motivated by a sub-strategy that seeks to align predicted with actual events. By this measure, each step in the action sequence is trying to resolve uncertainty about the next course of action by revealing hidden states in the world, drawing upon the wider action schema as an adaptive strategy when perfect alignment of predictions and events is unsuccessful. Time perception is the felt strain of the action sequence from beginning to end, in addition to the added strain of necessary adaptive behavior. Time perception can therefore fluctuate in response to this adaptation, influencing intensity, urgency, and the potential point of resignation along the action sequence.

### Questioning the Axioms of the Expected Utility Hypothesis

This of course questions one of the fundamental laws of expected utility theory, namely, the law of transitivity, since subjective value, and therefore preference, no longer can be said to be a stable property of any individual, for any duration of time. Moreover, it is also the case that the vast majority of preferred future states are themselves hidden states to be discovered through action, resulting in satisficing behavior (Simon, 1956), in many instances purely epistemic, i.e., novel, curiosity satisfying or salient. Few would therefore view a car as nothing but a machine that transports one from A to B, or food as a nothing more than simple sustenance. These things are imbued with properties to be discovered beyond their immediate function, which means that satisfying the same want can come in a myriad of different shapes, sizes, colors, textures, and flavors, leaving plenty of room for adaptive actions to solve the problem of how to satisfy a felt want.

In spite of the fact that a wide range of different goods has the ability to satisfy the same want, it cannot be said, however, that these goods therefore are of equal subjective value. Different action sequences (policies) imply different evaluations based on the different time strains they incur. This means that even two identical goods can be valued differently at different points in time depending on the amount of adaptation that is necessary in order to obtain or use the goods. Thus, even the truism of the reflexivity axiom cannot be said to hold when factoring in any length of time. Having to adapt policies during action sequences must hereby influence subjective valuations in numerous obvious and subtle ways, meaning that one of the staples of microeconomic consumer analysis, the indifference curve, is likewise not commensurate with any notion of choice in reference to human action and behavior.

This of course also questions the continuity and the revealed preference axiom. We can see this more clearly when considering that the suppression of variational free energy can be interpreted as a proxy for subjective value, where the factors that influence the former inform the latter. Thus, any attempt to measure or express the latter, will likewise encounter the problem of measuring or expressing the former, bearing in mind that variational free energy is an energy term and not a quantity. Thus, by optimizing beliefs, we remove the extensive property of utility and place it within a softmax function that is constantly being modified by the input from information gain. We can therefore say that monotonicity or positive affine transformations with respect to utility cannot apply to situations where there is uncertainty, since uncertainty resolution is the ultimate “purpose” of information gain, culminating in a variable time perception that alters whatever utility function that might be deemed representative of a stated set of preferences.

To illustrate this dynamic, imagine setting out to buy a long desired bicycle that now has come on sale. Arriving at the store you notice an unexpected queue forming outside. “Apparently you were not the only one interested in buying that particular bicycle.” Nevertheless, you must now decide whether to stand in line with the other customers or return home empty handed. Quite possible you will decide to wait in line, but the mere fact that you contemplated your options signifies a decrease in the present value of the bicycle, as it receded further into the future compared to your initial expected action sequence. Having now integrated your expectations with the act of standing in line, further frustrations or surprises might indeed prompt you to leave the store without completing your purchase, as the present value of the bicycle consequently would fall even further. However, you now spot an opportunity that allows you to skip waiting in line without being discovered by the other customers. Immediately you feel the intensity of your want increase and experience a heightened sense of urgency as your pace quickens and anticipation rises. Given your previous expectation (that included standing in line), the present value of the bicycle increases dramatically and adds an unexpected premium to the bicycle compared to earlier.

This dynamic places utility and choice behavior within a context that connects and mediates the expression of subjective utility through action and adaptation. As such, present value formulations become dependent on expected surprisal (risk), as well as unexpected surprisal (uncertainty), where utility changes dynamically in response to both. If an agent runs for the bus as it is about to leave station and is ascribing some probability for catching it, then he or she should be willing to pay a premium for the bus fare in proportion to the probability ascribed if actually catching it. In other words, an agent should be willing to pay a premium for successfully completing an action sequence when the action sequence affords the agent unexpected costs and should therefore also be willing to bear a relatively high unexpected cost in order to complete an action sequence. That this behavior occurs under an optimizing scheme puts irrational behavior such as the sunk cost fallacy in a new light, by highlighting some fundamental issues pertaining to a system that tries to make predictions in a fundamentally uncertain setting.

### Behavioral Implications of Indifference

The indifference curve describes a situation where an agent chooses between various configurations or bundles of two different goods, illustrated by a downward sloping convex curve. The convexity of the indifference curve is due to a diminishing marginal rate of substitution between the two goods, indicating that agents would prefer a mix of goods instead of a higher quantity of just one good. Any point on the curve is thus equally as preferential as any other point, allowing for the aforementioned indifference to occur. However, because the present value of all goods depends on the perceived time flow incurred from specific action sequences, urgency, and therefore preference, along with derived utility, shifts in response to any and all changes along the action sequence. This includes having to choose between bundles X and Y. Concordantly, there can be no indifference between one choice and another. This is partly because this would imply having to perform two mutually exclusive actions at the same time, but more importantly, because adaptation during any action sequence is unavoidable and that it would be correct to view this adaptation as either a gain or a loss function on the end goal in response to any and all dynamic updates to any action sequence (accumulated cost). A gain is when an unexpected opportunity presents itself, shortening time perception, heightening urgency, and increasing present value of the good toward which the opportunity was afforded, and a loss when externalities frustrate expectations, lengthening time perception, lowering urgency, and decreasing present value of the good toward which opportunity was frustrated.

The crucial part is that the initial act must be based on an established belief system (action schema) including a policy for resolving uncertainty. This expectation term selects an action sequence born from experience and, in so doing, posits a more or less articulated goal conveying a direction and a measure of time denoting urgency. Unless this expectation term is perfectly satisfied during the proposed action sequence, meaning that no new information can present itself, there is no reason to assume that setting out to buy milk will in fact result in the purchase of milk, to say nothing of a specific brand of milk. That is not the interesting part, however. The interesting part is that all new information is weighted against the expectation term, generating a modified action sequence based on the available responses/actions “stored” in the wider action schema. A necessary condition for indifference can therefore only be inaction, since every choice an agent could possibly make lowers the present value of every other alternative choice. If an agent proclaimed to be indifferent between a red shirt and a blue shirt, the only way to demonstrate this indifference would be for the agent to choose neither and walk away. If an agent chooses the blue shirt, but still proclaimed to be indifferent between the blue and the red shirt, then the agent should have no quarrels with swapping the one for the other. Also, once this swap was completed, the agent should not have any objections to swapping back. In fact, if the agent chooses the blue shirt, but simultaneously proclaims to be indifferent, then the agent should have no quarrels with swapping between the two shirts indefinitely. What breaks this endless loop can be cast in terms of variational free energy. When an action sequence is resolved, free energy is minimized. Expending further energy toward the resolution of the same want beyond this point introduces a new action sequence that cannot but increase free energy if the connected reward does not justify the computational complexity incurred save for belief updating (recall that risk corresponds to the expected complexity in Eq. 3). We see here that a choice once committed to must carry a premium over alternatives and will result in a different valuation behavior pre commitment compared to post commitment/choice. The literature on behavioral economics bears this out in numerous examples, the most prevalent of which is the endowment effect and the status quo bias (Kahneman et al., 1991).

Solving for preference is thus an “either/or,” not an “and/or” problem, meaning that there is only one solution and one solution alone to how the individual selects a given action sequence. Ignoring opportunity costs for the time being (i.e., money), giving up some of good X in order to get some of good Y is framing the problem incorrectly, since good X and Y are linked to two different policies that in many cases just happens to utilize a lot of the same actions up to a point. In instances where an individual does in fact substitute one good for another, this has to represent a decrease in the present value of the initial formulation, due to either an unexpected opportunity or an unexpected frustration. Invoking a probability *p* ∈ [0,1] that renders an agent indifferent between the lottery *pX* + (1−*p*)*Z* and the certainty of Y only serves to specify the point at which there can be no information gained through further action, and therefore, the point at which further action becomes meaningless. Action is inherently uncertainty resolving (Eq. 4). If an agent is indifferent between two outcomes, then the agent is not resolving uncertainty and is therefore not acting.

## Conclusion

This paper has used the variational free energy principle in order to describe how complex organic systems optimize while simultaneously being free to violate the axioms of expected utility theory. The approach is centered on the optimization of beliefs rather than the maximization of utility and, by this process, depicts rational behavior as following Hamilton’s principle of least action. This highlights the costs associated with deviations from least action and establishes choice behavior as a function of uncertainty resolution and adaptation (i.e., context). Once this is done, many behavioral “anomalies” become “rational” when framed in terms of a system that tries to make predictions and perform actions in a fundamentally uncertain setting.

Additionally, this has consequences for temporal discounting behavior, where discount functions become more or less erratic reflecting, and depending on, real time adaptations. This erratic discounting behavior can, however, be mitigated and mediated by the perceived uncertainty and expected complexity connected to state transitions over time.

However, the implication that agents do in fact optimize, and therefore do not exclusively employ psychological heuristics in the decision process, serves to restrain more extraordinary behavioral criticisms of standard economic theory. This also places the behavioral economist at a disadvantage when prescribing means and measures that purports to rectify apparent failings of human cognition and decision making.

## Data Availability Statement

All datasets generated for this study are included in the article/supplementary material, further inquiries can be directed to the corresponding author.

## Author Contributions

The author confirms being the sole contributor of this work and has approved it for publication.

## Conflict of Interest

The authors declare that the research was conducted in the absence of any commercial or financial relationships that could be construed as a potential conflict of interest.
